# Exploring the Piezoelectric Properties of Bismuth Ferrite Thin Films Using Piezoelectric Force Microscopy: A Case Study

**DOI:** 10.3390/ma16083203

**Published:** 2023-04-18

**Authors:** Denis Misiurev, Pavel Kaspar, Dinara Sobola, Nikola Papež, Saleh H. Fawaeer, Vladimír Holcman

**Affiliations:** 1 Department of Physics, Faculty of Electrical Engineering and Communication, Brno University of Technology, Technicka 2848/8, 616 00 Brno, Czech Republic; 186446@vut.cz (D.M.); sobola@vut.cz (D.S.); papez@vut.cz (N.P.); holcman@vut.cz (V.H.); 2 CEITEC BUT, Brno University of Technology, 612 00 Brno, Czech Republic; saleh.hekmat@ceitec.vutbr.cz

**Keywords:** multiferroic, nanomaterials, ferroelectric, bismuth ferrite, thin film, XPS, AFM, PFM, PLD

## Abstract

Over recent decades, the scientific community has managed to make great progress in the theoretical investigation and practical characterization of bismuth ferrite thin films. However, there is still much work to be completed in the field of magnetic property analysis. Under a normal operational temperature, the ferroelectric properties of bismuth ferrite could overcome the magnetic properties due to the robustness of ferroelectric alignment. Therefore, investigation of the ferroelectric domain structure is crucial for functionality of any potential devices. This paper reports deposition and analyzation of bismuth ferrite thin films by Piezoresponse Force Microscopy (PFM) and XPS methods, aiming to provide a characterization of deposited thin films. In this paper, thin films of 100 nm thick bismuth ferrite material were prepared by pulsed laser deposition on multilayer substrates Pt/Ti(TiO_2_)/Si. Our main purpose for the PFM investigation in this paper is to determine which magnetic pattern will be observed on Pt/Ti/Si and Pt/TiO_2_/Si multilayer substrates under certain deposition parameters by utilizing the PLD method and using samples of a deposited thickness of 100 nm. It was also important to determine how strong the measured piezoelectric response will be, considering parameters mentioned previously. By establishing a clear understanding of how prepared thin films react on various biases, we have provided a foundation for future research involving the formation of piezoelectric grains, thickness-dependent domain wall formations, and the effect of the substrate topology on the magnetic properties of bismuth ferrite films.

## 1. Introduction

In the world of an ever-increasing need of energy-efficient multifunctional material, the focus of the research lends itself towards new materials with different combinations of properties. Multiferroic materials attract considerable attention due to their unique combination of simultaneous magnetic and ferroelectric response [1].

Bismuth ferrite is a unique ferroelectric material due to its coexistence of ferroelectric and antiferromagnetic properties at room temperature. This characteristic arises from the complex spin structure of bismuth ferrite, which includes canted antiferromagnetic magnetic and weak ferromagnetic components. The coupling between ferroelectric and magnetic order parameters in bismuth ferrite is strong and originates from the underlying spin structure.

Among all potential candidates, bismuth ferrite stands out as a multiferroic material with the strongest electromagnetic coupling. The resulting magnetic spin orientation of bismuth ferrite material leads to formation of a weak magnetic spin moment and, as a result, huge spontaneous remanent polarization in range of ~90–100 μC cm^−2^. Bismuth ferrite is a multiferroic material with asymmetric rhombohedral perovskite structure with R3c space orientation with lattice parameters *a = b* = (5–7) Å and hexagonal *c* = (13–14) Å. The angle *α* is around 60 degrees [1]. Aside from having the unique combination of a magnetic and ferroelectric response, the material shows outstanding temperature stability with a high Curie [2] (*Tc* = 1120 K) point. The bismuth ferrite has a complex magnetic structure [3] due to interaction between its magnetic and electric dipoles [4]. The material exhibits antiferromagnetic behavior at room temperature with a Neel temperature *T_N_* = 650 K [5]. Bismuth ferrite’s magnetic structure displays a cycloidal spin arrangement in its bulk form, but in thin films, it can exhibit either canted antiferromagnetic or ferromagnetic behavior. The spin cycloid of bismuth ferrite refers to the periodic spatial vibration of magnetic moments. In bulk BiFeO_3_, the spin orientation of the magnetic domains is represented in cycloidal ordering, which is associated with a helical rotation pattern. The magnetic domains in BiFeO_3_ can demonstrate different spin orientations depending on the crystal structure and the direction of the applied magnetic field. The net magnetic moment of bismuth ferrite arises from the canting of its magnetic domains. These domains are separated by domain walls. The domain walls of bismuth ferrite refer to interface between the regions of the material that have a different ferroelectric domain orientation. These walls are characterized by the orientation of spontaneous polarization vector. The domain walls in BiFeO_3_ can have different morphologies including simple planar walls, curved walls, and more complex structures such as vertical or zigzag walls. The shape of domain walls is influenced by many factors, which would include surface morphology, indentations, crystallographic orientation, strain coefficient, and finally surface topology defects.

The discovery of bismuth ferrite as a single-phase multiferroic material has attracted significant attention in the scientific community, as its presence promises a potential pathway for the development of novel technological applications, such as sensors, memory devices, and spintronics. Moreover, this material is regarded as environmentally friendly due to its non-toxic nature and abundant constituents.

Despite the fact that the bismuth ferrite has been one of the most widely studied ferroelectric materials, there are other potential candidates that are worth mentioning. Most of them are inorganic materials: TbMnO_3_ (terbium manganite) [6], YmnO_3_ [7], BaTiO-CoFe_2_O_4_ (barium titanate-cobalt ferrite) [8], Pb(Zr,Ti)O_3_-Ni (lead zirconate titanate-nickel) [9], Cr_2_O_3_ (chromium oxide) [10], and N-methyl-4-nitroaniline (MNA) [11], (Bi0.85La0.15)FeO_3_-δ (labdanum doped BiFeO_3_) [12]. Every material exhibits simultaneous ferroelectric and antiferromagnetic properties, and each of them has the potential to be used in the wide spectrum of various applications and techniques, especially in the field of magnetic and electric sensors and spintronics. The PFM method has been intensively used in order to investigate ferroelectric properties, domain structure, formation of domain walls, and the hysteresis switching capabilities of these materials. In recent years organic ferroelectric materials started to gain popularity and have become a relatively new class of materials classified as inorganic–organic hybrids that exhibit both ferroelectric and magnetic properties. Some examples of modern organic multiferroic materials would include: β-Cu_2_V_2_O_7_·2.5H_2_O, Mn(TCNQ), Fe(phen)_2_(NCS)_2_, CH_3_NH_3_Mn(HCOO)_3_, [(CH_3_)_2_NH_2_][Mn(HCOO)_3_], and CuCrP_2_S_6_. These inorganic–organic hybrid materials are still at the early stage of research and development, but they increasingly show promise for a variety of technological applications.

Among the different types of ferroelectric materials, Pb-based piezoceramics such as lead zirconate titanate (PZT), lead magnesium niobate-lead titanate (PMN-PT), and lead zinc niobate-lead titanate (PZN-PT) have attracted significant attention due to their superior properties and industrial applications. PFM studies of PZT [13,14] revealed the presence of a variety of ferroelectric domains with different polarization directions and switching behavior under an applied external electric field. The domain structure of PZT is highly dependent on its composition, thermal history, processing condition, and crystallographic orientation [15]. PFM studies have also shown that the switching behavior of PZT is affected by the orientation of the crystal structure and the direction of the applied electric field. In addition, PFM studies have demonstrated the potential of PZT for using high-performance piezoelectric devices such as activators and sensors on transducers.

PMN-PT and PZN-PT are two other Pb-based piezoceramics that have attracted significant attention in the field of piezoelectric materials. Studies using PFM (Piezoresponse Force Microscopy) on PMN-PT [16] examine the presence of domains with multiple variants and the changes in the domain structure in response to temperature and electric field. Moreover, PFM studies of PMN-PT [17] have provided insight into the mechanical and polarization switching and the influence of crystallographic orientation on the domain structure. Similarly, PFM studies of PZN-PT have shown the presence of hereditary domains and the evolution of the domain pattern with temperature and electric field. These studies have also highlighted the impact of the composition process, condition, and thermal history of the domain pattern and switching behavior of PZN-PT.

In recent years, there has been a growing interest in developing Pb-free piezoceramics as an alternative to Pb-based materials due to environmental concerns. PFM studies have shown the existence of ferroelectric domains and their switching behavior under an external electric field [18]. The domain structure of BT-based ceramics is highly dependent on the composition, operational conditions, and thermal treatment [18].

Considering the relatively high chemical volatility of bismuth atoms during the deposition process, it is necessary to find appropriate ways to improve the chemical stability of bismuth atoms [19]. An increased presence of bismuth atoms ensures a dramatic decrease in parasitic current. A proper way to ensure stability of bismuth atoms and enhance electromagnetic coupling is choosing a proper substrate for the deposition of thin films. The most well-studied substrate is based on multilayers of Pt/Ti(TiO_2_)/Si substrate [19]. Pt is an inert material with appropriate thermal expansion and stability. In this structure, Pt provides a perfect electrical contact and an overall chemical stability of the deposited bismuth ferrite layer. In addition, Pt enhances electromagnetic coupling of ferroelectric films since Pt does not react with ferroelectric material [19] and shows some similarity with lattice parameters of bismuth ferrite material. It has been found that the deposition of a Pt layer directly on the Si substrate leads to migration of atoms from the Si substrate into the Pt layer, thus oxidating and, consequently, disturbing the overall integrity of Pt layer [19]. To prevent the occurrence of migration of Si atoms, it is necessary to deposit an inner layer of a material, which will prevent migration and ensure the integrity of Pt layer. So far, the Ti(TiO_2_) provides perfect integration of the Pt layer and prevents migration of Si atoms. In recent years, multilayer substrates based on Pt/Ti(TiO_2_)/Si [19] have been utilized increasingly often, since they provide an excellent electrical connection and complete separation of ferroelectric film. Deposited bismuth ferrite thin films on Pt/Ti(TiO_2_)/Si [20] multilayer substrates demonstrate remarkably high remanent polarization of ~150 µC cm^−2^, as well as a tetragonal crystallographic structure (P4mm) [20]. The potential description of crystallization into tetragonal structure could be given by morphotropic phase boundary of lattice parameters, which occurs due to the epitaxial growth of thin film [20]. The Pt/Ti(TiO_2_)/Si substrate is an effective platform for the growth of high-quality bismuth ferrite thin films, a wide range of desirable properties for potential applications.

## 2. Sample Preparation

In this paper, samples of BFO material have been produced by Pulsed Laser Deposition (PLD). In its operational principle, the method uses a powerful laser to evaporate a small amount of targeted material. Evaporated particles then condensate on the substrate, creating a demanded orientation of the final film. The unique advantage of the technology is deposition of heterostructures with a high degree of stoichiometry (homogeneity). Another considerable advantage of the method is the ability for sequential change in the targeted material, which allows the deposition of multilayer heterostructures with a combination of different materials. The method also operates in a low range of temperature (<400 °C), which is vital considering the dependence of quality of produced films on operational temperature.

**Figure 1:**
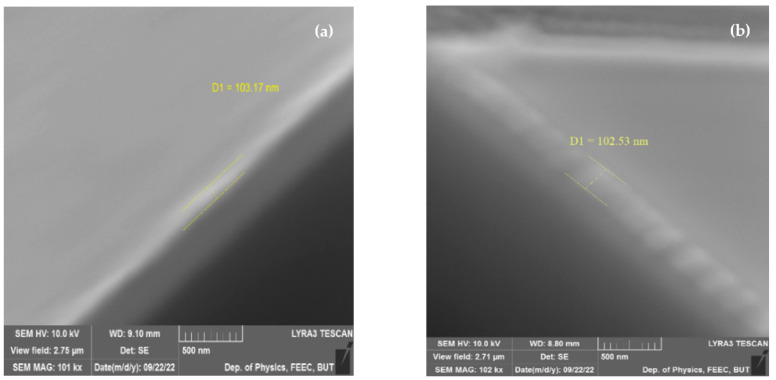
Cross section of 100 nm thick BFO samples (**a**) on Pt/TiO_2_/Si substrate (**b**) on Pt/Ti/Si substrate.

A total of 100-thick bismuth ferrite (BiFeO_3_) thin films were deposited onto Pt(10 nm)/TiO_2_(100 nm)/Si, Pt(10 nm)/Ti(100 nm)/Si substrates (Figure 1), and Pt(10 nm)/Ti(100 nm)/Si substrates, which were obtained by DC magnetron sputtering (BESTEC, Berlin, Germany) of Ti and Pt at room temperature by pulsed laser deposition (TSST, Strasbourg, France) at 530 °C and 7 × 10^–^³ mbar oxygen of partial pressure using a fluency of 2 J cm^−2^ for the excimer laser with a wavelength of 248 nm.

The BFO target was first cleaned with a pre-ablation of 2000 pulses at a repetition rate of 5 Hz, then the shutter was opened and BFO was deposited onto the substrates using 5000 laser pulses at a repetition rate of 5 Hz. A detailed description of deposition conditions of BFO thin films is listed in Table 1.

Produced samples of BFO material underwent scanning electron microscope in order to determine the degree of homogeneity of produced films on a cross section. Both scanning and cross section were performed on a Lyra 3 (Tescan, Brno, the Czech Republic) electron microscope with Ga FIB (Figure 1).

Thin films of bismuth ferrite deposited by pulsed laser deposition can exhibit various morphology defects, such as surface cracks indentations, and delamination. These defects can arise due to various factors, including substrate surface preparation, laser fluence, deposition temperature, and oxygen partial pressure.

The observation of produced 100 nm thick BFO samples indicates high degree of homogeneity of produced samples. However, some minor defects of surface are still precented.

**Figure 2:**
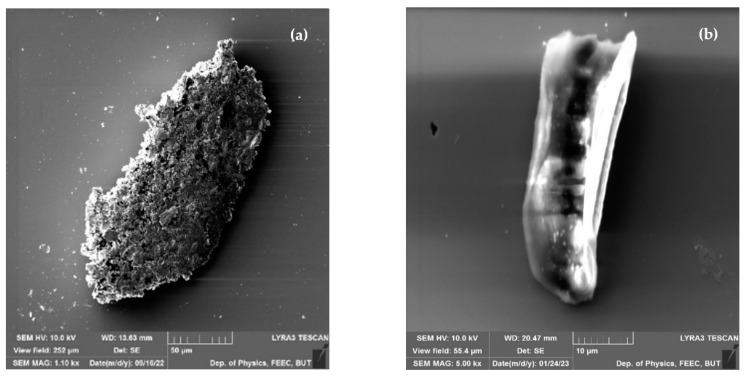
Artefacts on the surface (**a**) scanning surface (**b**) different scanning area.

Some surfaces of samples are covered by several artifacts (Figure 2) and small indentations, which could be related to an uneven layout of surface tension potential. Indentation can result from thermal stress during the cooling process, a difference in the thermal expansion coefficient between the film and the substrate, or gas bubble formation during deposition. Delimitation can occur due to a lack of interfacial adhesion or excessive residual stress in the field.

**Figure 3:**
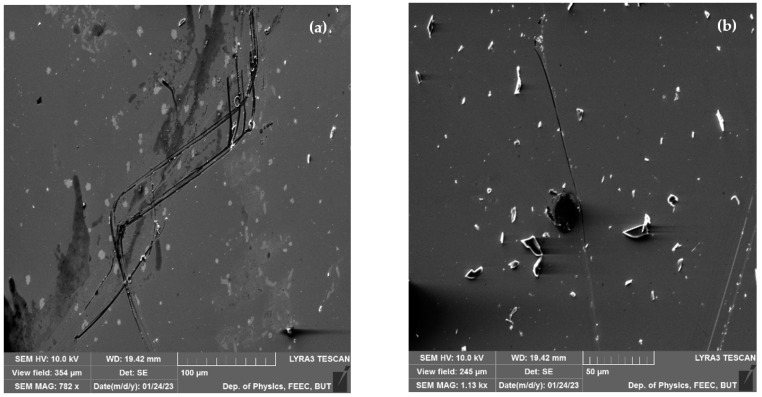
Mechanical scratches on surface of produced samples (**a**,**b**) different scanning areas.

Some of the samples show traces of mechanical deformation, which could be attributed by manipulation (Figure 3), or it can be attributed to poor adhesion on the film to the substrate surface, insufficient heating, cooling stress, or inhomogeneous deposition rate. In addition to mechanical deformation, Figure 2a also demonstrates negligible traces of contamination or delimitation, which can occur due to a lack of interfacial adhesion or excessive residual stress in the field.

On the other hand, surface of the samples is covered by contamination islands, but even after intensive cleaning by compressed air these islands remain on the surface of samples (Figure 3b). As it has been mentioned, these islands could be attributed to clusters of the BFO material, which occur during epitaxial grow of initial thin film on top of the multilayer substrates.

To minimize any potential defects, optimization of deposition parameters including substrate, surface preparation, laser parameters, and post-deposition annealing conditions.

## 3. Results and Observation

### 3.1. X-ray Spectroscopy Analysis

X-ray photoelectron spectroscopy (XPS) is a straightforward process, which has gained a lot of popularity and is widely used for kinetic electron emission techniques. The physical principle is based on the photoelectron scattering effect and binding energy. In the case of bismuth ferrite, XPS can be used to absorb the effect of spin orbit interaction on the electronic state near the surface of the material. By analyzing the binding energies and line shapes of XPS spectra, it is possible to extract information about the spin–orbital split level and the magnetic properties of the sample. This can provide information regarding the role of spin–orbit interaction in determining physical properties of bismuth ferrite. Spin–orbit interaction in bismuth ferrite refers to the coupling between the intrinsic angular momentum of an electron and its motion in an epitaxial potential. This interaction results in a modulation of the energy levels and the magnetic properties of the material. The spin–orbit interaction in bismuth ferrite is given by presence of heavy elements such as bismuth and iron and has been shown to play a crucial role in its magnetic and ferroelectric properties. The main purpose, for which the method is extensively used in many applications, is mainly the determination of the chemical composition of the material. Additionally, XPS can be used to study the effect of surface modulation and effects on the electric structure of bismuth ferrite. The XPS spectrum was obtained by KRATOS XPS spectrometer with X-ray Power 225 W and emission current 15 mA of sample with surface thickness being approximately 100 nm.

**Figure 4:**
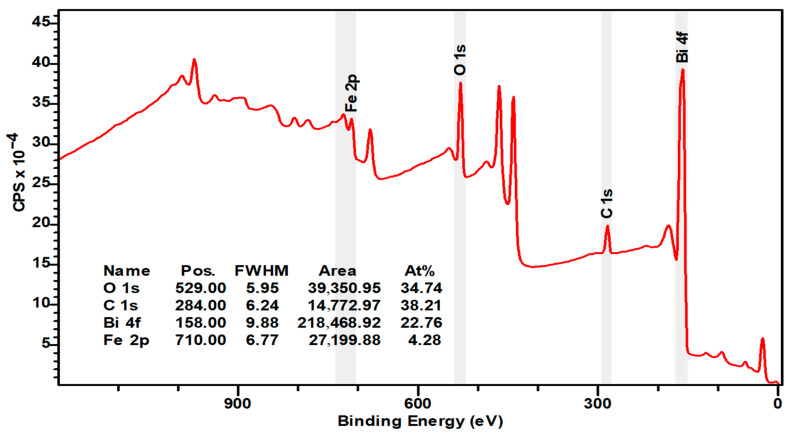
Wide XPS spectrum of 100 nm BFO (Pt/TiO_2_/Si) sample.

**Figure 5:**
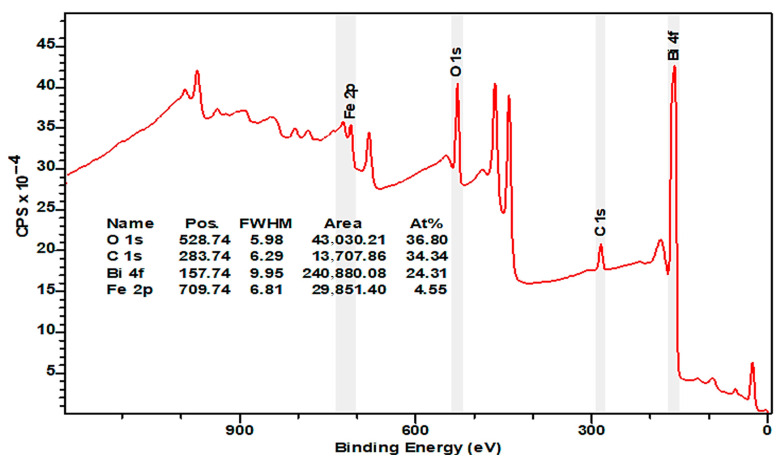
Wide XPS spectrum of 100 nm BFO (Pt/Ti/Si) sample.

Figure 4 and Figure 5 represent wide X-ray spectra from 0 to 1000 eV. The wide spectrum identifies molecular composition of presented chemical elements. The spectrum shows typical chemical elements of a BFO sample, which are oxygen, carbon, iron, and bismuth.

**Figure 6:**
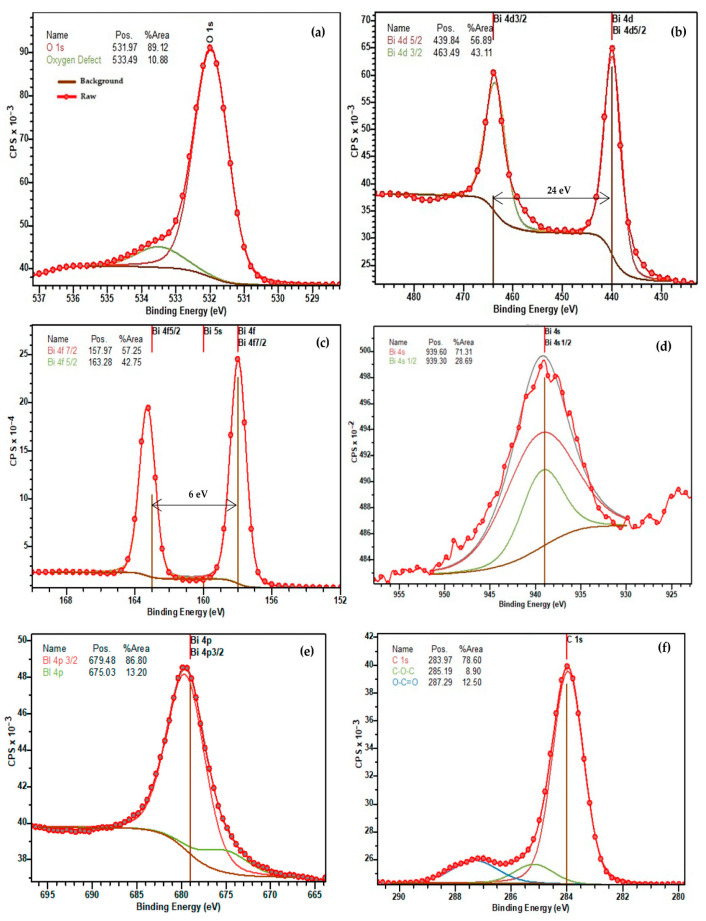

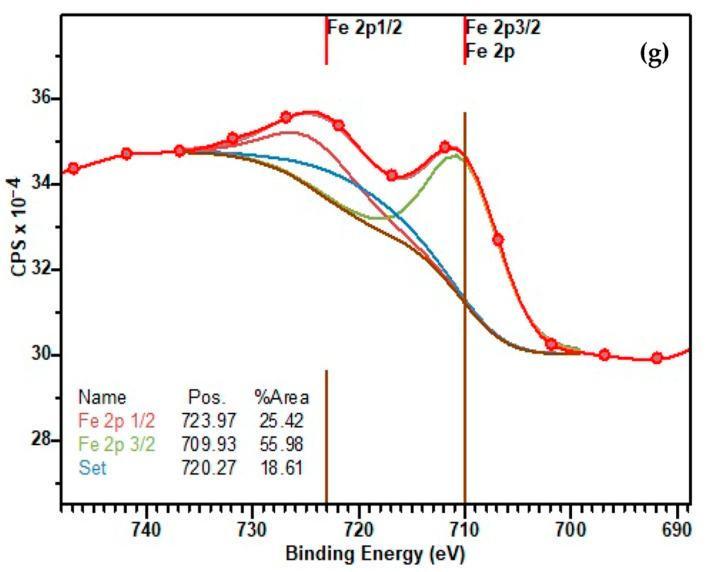
XPS spectra of single element (**a**) O 1s; (**b**) Bi 4d; (**c**) Bi 4f; (**d**) Bi 4s; (**e**) Bi 4p; (**f**) C 1s; (**g**) Fe 2p.

**Figure 7:**
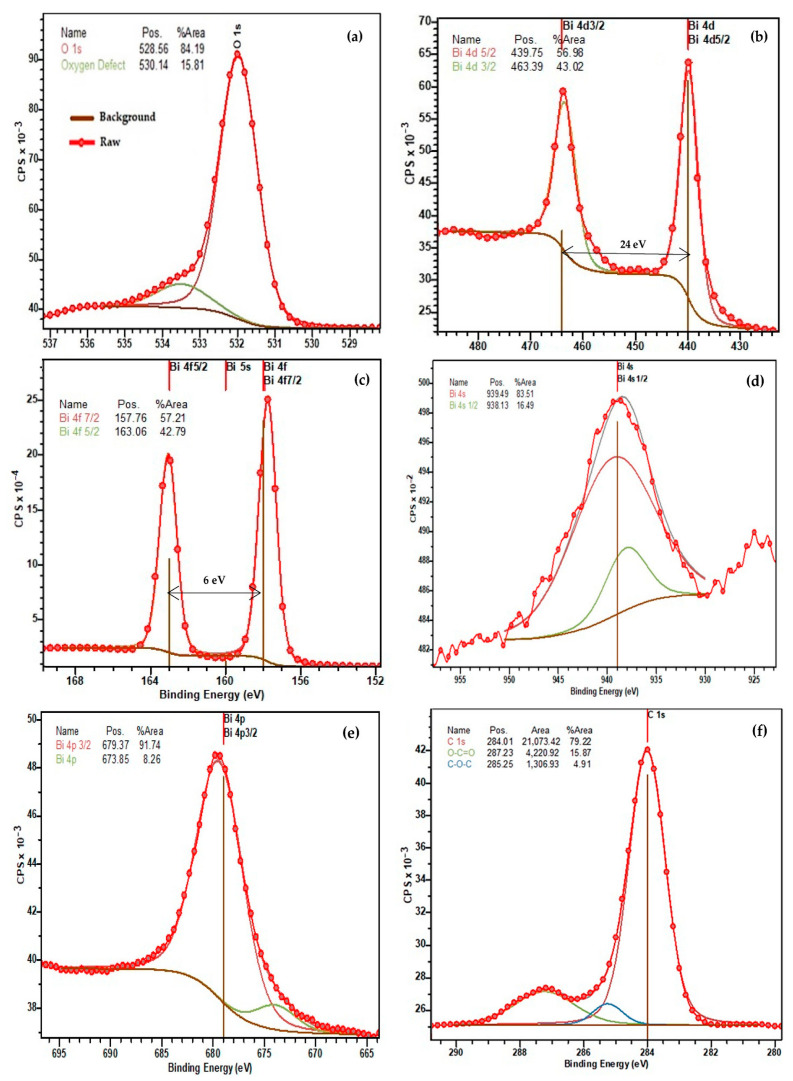

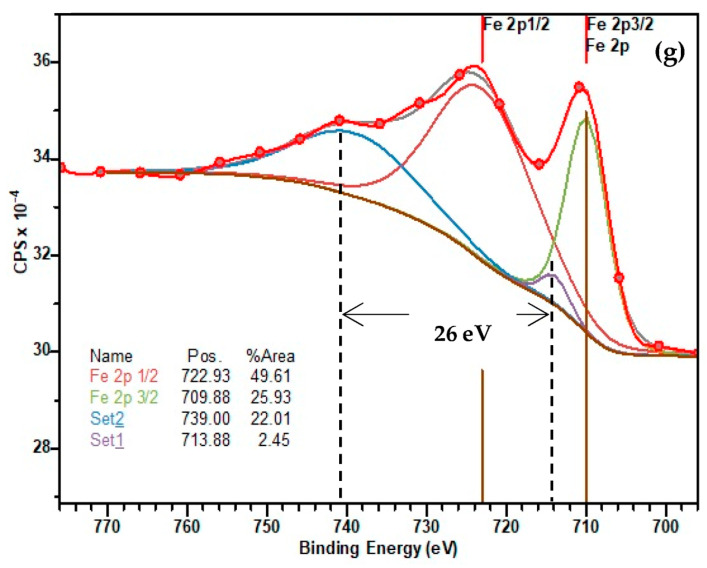
XPS spectra of individual elements (**a**) O 1s; (**b**) Bi 4d; (**c**) Bi 4f; (**d**) Bi 4s; (**e**) Bi 4p; (**f**) C 1s; (**g**) Fe 2p.

The carbon peak (C 1s) is observed at a binding potential of 284 eV (adventitious carbon), which is a result of surface contamination from exposure to air. This peak is primarily utilized for compensating charging effects and calibration purposes. The carbon content was diminishing with longer etching. Peaks with a binding energy of 285 and 287 eV are associated with C–O–C and O–C=O molecular bonds (Figure 6f and Figure 7f). Binding energy peaks at 159 and 164 eV were observed for Bi 4f _7/2_ and Bi 4f _5/2_, respectively, with an energy difference around 6 eV between peaks (Figure 6c and Figure 7c). The energy of Bi 4f _7/2_ corresponds with Bi^3+^ chemical valency and the oxygen bond [21]. The stronger signal in Bi 4f is attributed to its deeper cross-section and the presence of a bismuth carbonate compound, which was formed during X-ray analysis. In addition to Bi 4f _7/2_ and Bi 4f _5/2_ doublet, XPS spectrum indicates a strong presence of oxygen atoms [22]. Both Bi 4f and O 1s photoelectrons are a part of the BiFeO_3_ surface. The binding energy of O 1s stays within range from 525 to 540 eV (Figure 6a and Figure 7a), which corresponds with chemical bond of oxygen atoms with metal atoms such as bismuth or iron [22]. The presence of O 1s photoelectrons confirm the absence of the secondary parasitic phase. However, there is a small amount of oxygen contamination, which can be seen in Figure 6a and Figure 7a. An XPS spectrum also indicates strong presence of Bi 4s and Bi 4s _1/2_ doublet with binding energy 939 and 938 eV (Figure 6d and Figure 7d).

Besides C, Bi, and O elements, the XPS spectrum also indicates presence of pure Fe and Fe^3+^ content at 709–727 eV on the surface, which could be potentially be related to the parasitic compound of Fe_3_O_4_ oxide [23]. Fe 2p _3/2_ and Fe 2p _1/2_ [24] both correspond with Fe ^2+^ and can be observed on the spectrum (Figure 6g and Figure 7g) [25]. The satellite peaks were observed on both spectra (Figure 6g and Figure 7g). Nevertheless, the secondary satellite peak was absorbed only on one spectrum (Figure 7g). The energy difference between satellite peaks is around 26 eV. The Fe 2p spectra suggest a high degree of symmetry, but they are still asymmetric, likely due to the formation of oxygen vacancies. Changes in chemical valency of Fe^+3^ to Fe^+2^ [26] is because of these vacancies. The presence of satellite peaks is associated with Fe^+3^ [27], which are represented in the form of an oxide. The location of satellite peaks can be seen on both spectra. A high degree of symmetry of both peaks of Fe^+2^ is given by a small amount of the formation of oxygen vacancies [28]. Both the high content of Fe^+3^ and the low presence of oxygen vacancies would have a dramatic effect on overall magnetic properties of the overall cell unit therefore enhancing them. Energy peaks are shifted towards carbon compounds. There is little difference between the two samples, considering the presence of a Pt monolayer, which will prevent any potential diffusion into BFO layer. In addition to the Pt monolayer, titanium oxide and titanium will provide an outstanding integration of Pt and other monolayers including the Si substrate.

### 3.2. Piezoresponse Force Microscopy Analysis

The Piezoresponse Force Microscopy (PFM) method is based on the detection of displacement which is caused by a variable electric field [29]. PFM is mainly used for evaluation of ferroelectric materials and provides characterization of electromechanical behavior in terms of their domain orientation and polarization. In addition to characterizations of electromechanical behavior, common PFM applications include testing all sorts of microelectronic devices, electro-optical, and memory devices by investigating their reliability to withstand stress [29].

The principle of the method is based on applying AC Bias (1) between the investigated sample and bottom electrode (sharp tip) to provoke a local piezoelectric response, which then triggers local distortion [30].

V_tip_ = V_dc_ + V_ac_ cos (Ωt + φ) (1)

By utilizing look-in technology detection and recording the piezoelectric vibration, characterization can be performed in order to determine orientation of polarization within a thin film.

The local distortion is then captured by the cantilever (deflection (2)), which is given by equation:

D = D_0_ + A (w, V_dc_) + V_ac_ cos (Ωt + φ) (2)

The total piezoelectric displacement of magnetic domains is a vector which is based on the direction of the AC bias and can be divided into vertical and lateral components, also referred to as in-plains and out-of-plane. Their orientation is given by a different crystallographic orientation of grains. Out-of-plane domain stands for normal spontaneous polarization direction, which, in turn, brings up vertical vibration of the surface and, thus, cantilever. Out-of-plane domain polarization associates with a positive direction of *y*-axis [30].

In-plane domain polarization, on the other hand, is distributed within the surface of the film and shares oscillation, which is detected by cantilever. In-plane domain polarization is orientated with a negative direction of *y*-axis [30]. However, polarization vector of ferroelectric domains alongside with cantilever axis does not indicate any in-plane domain polarization contrast [30].

The PFM images represent switching behavior between perpendicular components of polarization, which can be divided into two states: dark and bright. The bright region corresponds with downward direction of polarization, which means that the polarization vector is oriented downward and associated with the [111] or [1–11] polarization direction [30]. Dark regions, on the other hand, are oriented upward and related [−1–11] or [−111] direction [30].

The difference between in-plane and out-of-plane domain polarization can be explained by the fact that the response direction of in-plane polarization remains unchanged upon switching, whereas the out-of-plane polarization does respond [30]. Another difference is the antiferroelectric 180° switching direction, which can occur through different ferroelastic angles (71° and 109°) and is related to a single-component switch of electrical polarization [30].

By comparison of the sin-plane and out-of-plane response, the reconstruction of a 2D Piezoresponse map of polarization distribution can be performed. Main attributes, such as angle (phase) and magnitude maps, can be reconstructed from the 2D vector Piezoresponse map. Phase and magnitudes images would provide comprehensible representation of structure and orientation of each domain. The phase map indicates a difference in polar orientation of the domains [31].

The multiferroic behavior of BFO films can be manipulated by electrical potential, which is related to ferroelectric switching. In order to create local switching, AC potential is applied to a conductive sharp probe during the scanning procedure. Thus, analyzing in-plane and out-of-plane changing orientation followed by electrical poling, a basic switching mechanism will be detected. These mechanisms are related to different angles (71°, 109°, and 180°) [31], under which switching will occur. Single angles are related to different switching events. Angles 71° and 109°, in most cases, are related to a ferroelastic switching mechanism, where remaining domains (180°) show a ferroelectric response [32]. A switching mechanism with 180° angles is represented as a reversal image coloration in both in-plane and out-of-plain directions [32]. A ferroelastic 109° mechanism is given by reverse change in out-of-plane image coloration [32]. However, there is no change in in-plane coloration image.

Most of PFM measurements are conducted in order to create a map of a domain wall structure of ferroelectric material. Domain walls are interfaces between regions of the material that have different ferroelectric domain orientations. One of the key features of domain walls is the rotation of polarization vectors across the interface. This rotation is mediated by the presence of the defects in the strain fields, which act to lower energy domain wall formation. Magnetic materials typically exhibit domain wall formation due to the presence of magnetic domains with different orientations. These domain walls have distinct properties compared to the bulk material [33]. The bismuth ferrite is represented by various types of domain walls, based on crystal structure grow conditions. The common type is the 90° domain wall [14,30], which separates regions with polarization vectors orientated perpendicularity. Another type of domain wall which is observed in bismuth ferrite thin films involves 180° [14,30] and 109.5° domain walls. The domain wall structure map reflects the direction of polarization of ferroelectric domains. The formation of domain walls is affected by many factors. The most widely spread factor, which causes a dramatic effect on domain wall formation, result from impurities in rare–earth materials [33].

Different lithographic structures affect surface tension potential, thus changing the conductivity and light absorption capability, which would be related to various orientations of dielectric relaxation and ferroelectric response [34]. PFM imaging shows the surface topography of the produced samples, which will include domain wall structure. Due to uneven surface morphology, which includes indentations, material clusters, and so on, domain structure transition exists. The existence of domain structure transition is given by reorganization of domain polarization, due to mechanical and electrical structural inhomogeneity [34].

**Figure 8:**
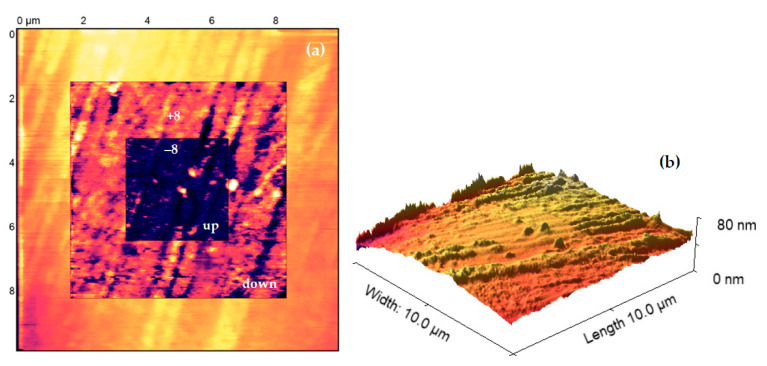
Topography of 100 nm thick BFO Pt/TiO_2_/Si (**a**) square pattern +/− 8 and 0 V bias (**b**) 3D topography.

**Figure 9:**
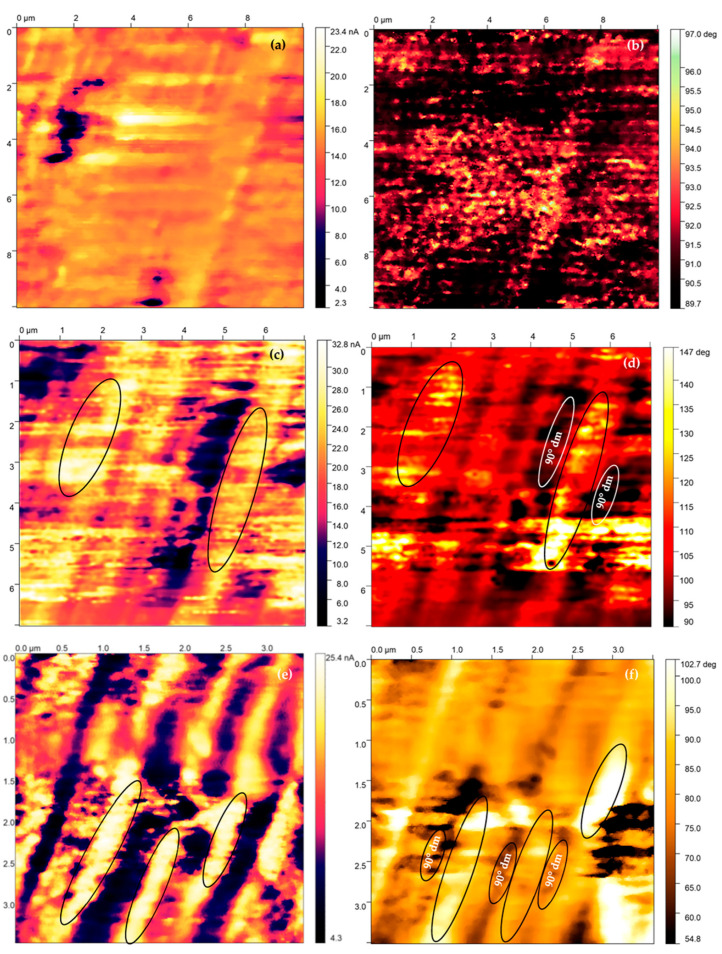
Current distribution and phase alignment for 0 V (**a**,**b**) −8 V (**c**,**d**) 8 V (**e**,**f**) bias BFO 100 nm Pt/TiO2/Si.

**Figure 10:**
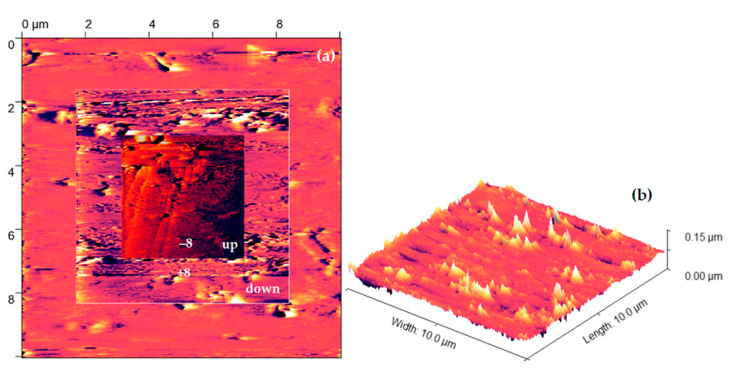
Topography of 100 nm thick BFO 100 nm Pt/Ti/Si (**a**) surface under +/− 8 and 0 V bias (**b**) 3D topography.

**Figure 11:**
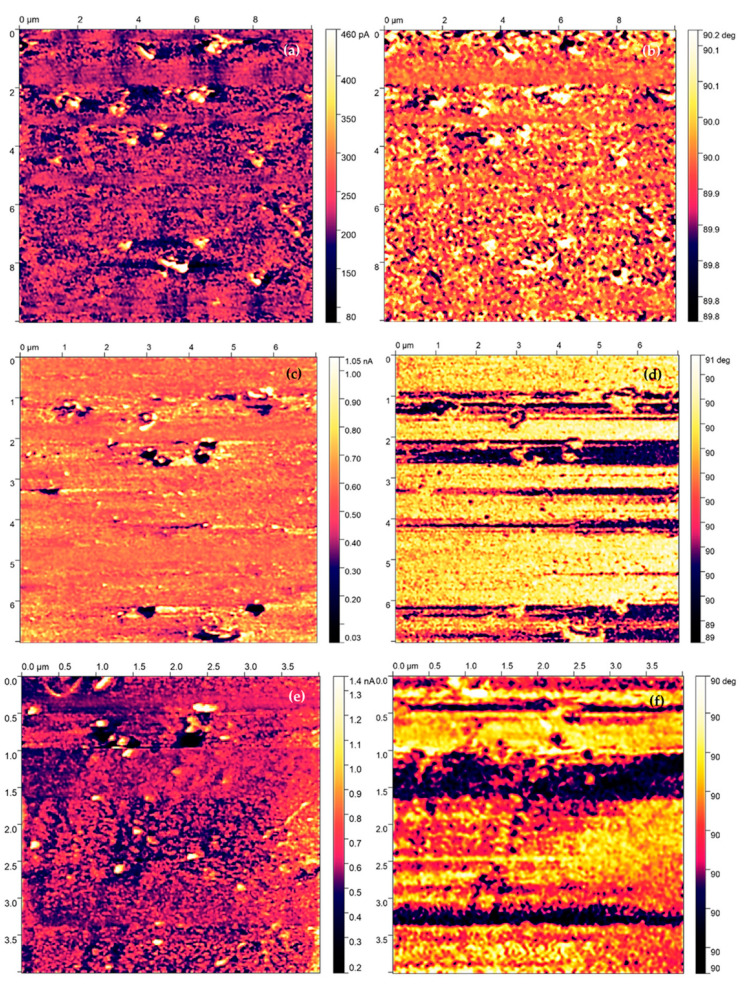
Current distribution and phase alignment for 0 V (**a**,**b**) −8 V (**c**,**d**) 8 V (**e**,**f**) bias BFO 100 nm Pt/Ti/Si.

The PFM measurements have been performed on NTEGRA Prima from company NT-MDT. FMG01/Pt sharp probe was used in order to perform PFM measurement. The surface of the produced samples was analyzed by using Piezoresponse Force Microscopy. The Figure 8, Figure 9, Figure 10 and Figure 11 indicate the surface of the analyzed sample 100 nm BFO (Pt/Ti(TiO_2_)/Si) samples, where the size of the investigated area of samples is (10 × 10) µm^2^. The measurement has been performed with a scanning frequency of 0.25 Hz to obtain current distribution and phase images (Figure 9 and Figure 11). The samples underwent PFM analysis under +8, −8, 0 voltage bias in order to determine the switching mechanism of the magnetic domains in the prepared samples (Figure 9 and Figure 11). In addition to the topography images, images of local current distribution and phase alignment are provided. A current image represents the distribution of the piezoelectric current generated in response to the AC voltage modulation, which is proportional to the local piezoelectric officiant of the deposited material (Figure 9 and Figure 11). Phase image represents the phase difference between the AC voltage applied to a sharp tip. The magnitude and polarity of the piezoelectric current are proportional to the piezoelectric coefficient and the direction of polarization of the sample. Both current image and phase image provide the mapping of the domain structure of the bismuth ferrite thin films, which reveal the orientation and distribution of piezoelectric active domains. A Gaussian filtering function was used to create more visible and pronounced piezo-active regions and magnetic patterns.

When the electric field is applied parallel to the direction of polarization, the ferroelectric material undergoes out-of-plane elongation, which results in a bright contrast in the current distribution image. By analyzing the samples under different a bias potential, the current image and phase image revealed that the produced samples of bismuth ferrite have a complex domain structure with a stripped-like patter alignment of domain moments, which is the most common type of domain formation. Types of domain formation are reduced by a strain relaxation difference related to the asymmetry of the in-plain images. Therefore, deposited samples of 100 nm thick BFO films demonstrate a stripped alignment pattern. Considering Kittel’s law, the energy of domains across the entire thickness of the sample is reversely proportional to sample thickness [35]. The elastic energy of the domains is less effected in thinner samples, preventing the different patterns to occur. The extremely bright coloration on current distribution and phase images illustrates highly ordered domain clusters and a switching process in the analyzed areas. The stripe pattern is created by two polarization vectors, which are aligned 71° apart. The direction of polarization vectors is orientated into the substrate. The 71° switch mechanism is related to ferroelastic domains and characterized by change only in the out–of–plain coloration image.

When an external electric field is applied to the ferroelectric material, the direction of its electric dipole movement can be reversed, resulting in a change in a polarization direction. In certain ferroelectric materials, a 71° switching mechanism exists where the polarization direction changes by an angle of 71° relative to the original direction. The 71° switch mechanism is facilitated by the formation and propagation of the domain walls, where the polarization direction changes abruptly (Figure 9b,d,f) [14]. In BiFeO_3_ thin films, 71° domain configurations give rise to the accumulation of boundary charges with the same site as the interface. This results in the generation of a significant depolarization field, which ultimately leads to the depolarization of the domain structure. The high conductivity of the deposited bismuth ferrite monolayer, which reflects the boundary charges, leads to the creation of an overly stable 71° domain structure. Figure 9e, f clearly indicates the predominant orientation of domains in a 90° angle, which would be attributed to a loss of elastic energy associated with the switching process (elastic recovering).

The PFM investigation of the second sample did not reveal a 71° switching mechanism. Nevertheless, the coloration of the investigated regions exhibits different contrasts under various applied voltage biases, which confirms the piezoelectric activities of investigated samples with correlation to the current distribution images (Figure 11a, c, e). The phase images of domain orientation reveal that the magnetic domains did not exhibit a uniform, well-defined, striped alignment of domain moments (Figure 11b, d, f). However, according to the phase images, the magnetic domains seem to organize into straight lines, which would suggest the creation of local but weak stripe patterns. Therefore, we speculate that there could be some issue related to the scanning probe of PFM, and that potential damage could have been sustained during previous scanning.

## 4. Discussion

Bismuth ferrite thin films have attracted significant attention in the scientific community to their unique properties, including ferroelectricity, ferromagnetism, and multiferroicity. The investigation of the magnetic properties of bismuth ferrite has resulted in the discovery of 71° domain walls. The discovery of this type of domain wall reveals new avenues for research in the field of domain wall physics.

To achieve superior performance of deposited bismuth ferrite thin films without any defects or dislocations, it is important to conduct a systematic investigation of the impact of thin film processing parameters on the surface chemistry of these heterostructures. Investigation can provide a valuable insight into the underlying mechanism in the formation of the magnetic patterns of thin films, thus creating and developing new strategies to optimize overall performance.

We were able to deposit bismuth ferrite thin films with a high degree of repeatability and consistent morphology, thanks to the flexibility of PLD and the low operational temperature. However, we could potentially encounter several problems related to the thickness of the deposited films, since as the film thickness increases, the properties of the film can become dominated by the surface effect rather than bulk effects. One of the main issues is the development of cracks and defects due to the internal stress caused by lattice mismatch or a difference in the thermal expansion coefficient, which could lead to dramatic decreases in film quality and magnetic properties. The thickness of the thicker films affects the domain walls weight. These domain walls may become wider, leading to a reduction in functionality. One of the challenges is the uniformity of the film thickness, which leads to inhomogeneity of the film thickness.

XPS spectra revealed strong presence of heavy elements such as bismuth and Iron and has been shown to play a crucial role in bismuth ferrite magnetic and ferroelectric properties. The presence of these materials gives rise to the spin–orbit interaction of the bismuth ferrite material.

One of the key elements of bismuth ferrite, which defines the magnetic properties of the material, is the presence of oxygen defects. The oxygen defects within the rhombohedral structure of the bismuth ferrite cell unit cause distortion of the antiferromagnetic order creating a weak ferromagnetic magnetic response. According to the analyzed XPS spectra, the produced examples exhibited around 15% defects of total concentration of oxygen, suggesting the presence of a weak ferromagnetic response. XPS spectra did not reveal the presence of secondary phases of bismuth ferrite or any potential parasitic elements contamination.

The PFM current images reveal a high piezoelectric current generated across the investigated area, which reflects the high piezoelectric activity of the produced samples. Under different applied voltage bias, investigated areas changed the coloration by changing the orientation of magnetic domains. As a result of this change, the magnetic domains form a strip domain pattern, which is the most common domain pattern in bismuth ferrite thin films. The phase images of PFM analysis revealed the magnetic domains of the deposited samples, demonstrating a 71° switching mechanism. The switched domains were divided by 90° domain walls.

Considering Kittel’s law, the energy of domains is proportional to sample thickness. It would be beneficial to determine the critical thickness, under which the bismuth ferrite thin films undergo transition to different switching mechanisms.

Current trends of development of bismuth ferrite thin films involve investigation and manipulation with the domain walls to provide understanding of their unique properties and reveal their potential. Since the topography of the deposited substrate greatly affects the properties of deposited thin films, it would be interesting to provide a study that focused on the investigation of magnetic and piezoelectric properties by utilizing a substrate with different morphology.

Our main purpose of the PFM investigation in this paper was to determine which magnetic pattern would be observed on Pt/Ti/Si and Pt/TiO_2_/Si multilayer substrates under certain deposition parameters. This was achieved by utilizing the PLD method and using samples with a deposited thickness of 100 nm. It was also important to determine and measure how strong the piezoelectric response was, considering the parameters mentioned previously. By establishing a clear understanding of how prepared thin films react on various biases, we provided a foundation for future research involving the formation of piezoelectric grains, thickness-dependent domain wall formation, and the effect of the substrate topology on magnetic properties of bismuth ferrite films.

## 5. Conclusions

Thin films of bismuth ferrite material were successfully prepared by pulsed laser deposition technology. Produced samples of 100 nm of BiFeO_3_ on Pt/Ti/Si and Pt/TiO_2_/Si substrates underwent different analysis methods in order to determine the behavior of piezoelectric.

X-ray spectroscopy produced samples of BFO material, which exhibited low contamination by carbon molecules, as well as low levels of oxygen vacancies. XPS spectra revealed the scattering peaks of bismuth atoms at different binding energies, which indicates a high concentration of bismuth material within a cell unit. Strong presence of bismuth gives rise to the spin–orbit interaction, which has been shown to play an important role in magnetic properties, resulting in large magnetic anisotropy and magnetic switching.

XPS spectra indicate the presence of oxygen defects, which cause a significant impact in relation to the magnetic properties of the deposited films. Considering the substantial noise signal and abdominal behavior demonstrated by bismuth ferrite, we could not identify and distinguish all peak of the constituent elements.

The PFM analysis revealed an extremely high response in piezoelectric activity across the investigated area, which confirms our speculation regarding sophisticated quality of bismuth ferrite films deposited by the PLD method. According to our observation, we determined that prepared thin films demonstrate strip patter alignment of domain moments with a 71° switching mechanism. The strip patterns are separated by 90° domain walls.

## Figures and Tables

**Table 1:** Deposition conditions of BiFeO_3_ thin films by PLD.

Substrate	Sample	Fluency (J/cm^2^)	Laser Pulses	Repetition Rate (Hz)	Deposition Time (min)	Substrate Temperature (C°)	Gas Pressure (O_2_) (mbar)
Pt(10 nm)/TiO_2_(100 nm)/Si	# BFO-100	2	5000	5	16.7	530	7 × 10^–^³
Pt(10 nm)/Ti (100 nm)/Si	* BFO-100						

* and # are part of the sample label.

## Data Availability

Data is available in Supplementary Materials.

## Supplementary Materials

The following supporting information can be downloaded at: https://www.mdpi.com/article/10.3390/ma16083203/s1.
